# Nutrition and Health Claims: Consumer Use and Evolving Regulation

**DOI:** 10.1007/s13668-022-00422-3

**Published:** 2022-05-24

**Authors:** Elizabeth P. Neale, Linda C. Tapsell

**Affiliations:** 1 School of Medical, Indigenous and Health Sciences, Faculty of Science, Medicine and Health, University of Wollongong, Wollongong, NSW 2522 Australia; 2 Illawarra Health and Medical Research Institute, Wollongong, NSW Australia

**Keywords:** Nutrition content claims, Health claims, Food regulation, Food labelling, Australia

## Abstract

**Purpose of Review:**

The value of nutrition and health claims (N&HC) depends on how consumers use them and the regulatory framework that enables them. This paper aims to explore the impact of claims on consumer behaviour and identify evolving regulatory challenges, using the Australian experience as a reference point.

**Recent Findings:**

N&HC can influence consumer food purchasing and consumption, but how consumers interpret and act on specific claims is less well understood, and regulatory frameworks are evolving. In the last 10 years, changes to the Australian regulatory framework have exposed greater opportunities for promoting foods, albeit with challenges regarding self-substantiation of claims.

**Summary:**

N&HC can play a significant role in driving consumer choices towards a healthier food supply. The Australian experience demonstrates how N&HC can continue to evolve, reflecting developments in methodologies and a fundamental appreciation of the relationship between food and health.

## Introduction

Understanding the impact of food consumption on health is not just the domain of scientists, it has significant implications for consumers, food producers and regulators alike. There is a common ground in communicating the benefits of foods to assist in food choices, but this is a complex area of varying cultures and priorities. From a nutritional health perspective, there are two main areas in which food consumption can provide benefit: delivering key nutrients to meet requirements, and supporting health (assisting normal growth and development, and protecting against chronic disease) [1]. The science that underpins these perspectives draws on research on nutrient action, nutrient requirements in health and disease and the relationships between dietary intakes and growth, development and disease [2]. These are complex areas that nevertheless require adequate translation and communication for all stakeholders in the health claims arena.

Where food packaging and advertising is concerned, nutrition and health claims (N&HC) cover a range of statements established by regulatory bodies within jurisdictions around the globe. These include the US Food and Drug Administration (FDA) [3], Health Canada [4], the European Food Safety Authority (EFSA) [5] and Food Standards Australia New Zealand (FSANZ) [6]. While there is some variation between the specific definitions and types of claims, as well as regulatory processes and requirements, a consistent characteristic is the presence of nutrition content claims, and one or more level of health claims. Reflecting the key benefits of food consumption, these statements are primarily gauged in terms of nutrient action and diet-disease relationships and are underpinned by scientific evidence derived from current nutrition knowledge. Thus, while precise definitions may differ between regulatory bodies, the purposes of assuring delivery of key nutrients, and addressing the relationship between food consumption and health/disease remain common. Claims typically relate to ‘nutrient content’, referring to the amount of a nutrient contained in a food product; and ‘health’ referring to either the effects of a nutrient on functions of the body or the dietary association with risk of disease [3–6]. It follows that Health Claims are typically further sub-categorised into general level claims on nutrient functions and processes (e.g. *calcium is necessary for normal teeth and bone structure* [7]) or high level claims based on reduction in disease risk (e.g. *a diet high in calcium with adequate vitamin D status reduces risk of osteoporosis in persons 65 years and older* [7]).

In Australia, the Food Standards Code outlines the nutrition content and health claims which can be made on food labels and in advertising, and the conditions by which they can be made [6]. While FSANZ is responsible for the Food Standards Code, health authorities at the state and territory levels are responsible for enforcement or monitoring of compliance with the Code, requiring co-ordination across state jurisdictions. Furthermore, aspects of the framework differ between the different types of claims. For nutrient content claims to be made, the foods must adhere to pre-specified conditions, e.g. meeting a defined level of the referent nutrient. For instance, to make claims on vitamin or mineral content, a serving of food must contain at least 10% of the recommended dietary intake [6]. Health claims address the more complex issue of the relationship between diet and health (in the case of general level health claims), and diet and disease (in the case of high level health claims), which goes beyond the simple delivery of nutrients. This relationship has been studied on a number of levels. One of the major challenges in this area is the recognised interdependence between nutrients, foods and dietary patterns [1]. Nutrients are delivered by foods, and the combination of foods (dietary patterns) ultimately influences health outcomes [1]. The evidence to date is that, chronic disease is linked to dietary patterns that exceed energy requirements, and contain high levels of saturated fats, sodium and added sugars. On the other hand, dietary patterns rich in vegetables, fruit, nuts and with adequate levels of fibre and protein appear protective [8, 9]. A practical translation of this knowledge is the Nutrient Profiling Scoring Criterion (NPSC), used to evaluate foods based on a range of components judged as ‘negative for health’ (energy, saturated fat, total sugars and sodium) and ‘positive for health’ (fruit, vegetables, nuts, legumes, fibre and protein). Thus, general or high level health claims can only be applied for foods with a score below a set cut-off [6]. While there are obvious limitations in applying dietary pattern–based evidence to single foods, it is a reasonable assumption that if ‘negative’ nutrients are discouraged, the total diet will benefit.

In continuing to refine the science and its translation, nutrition scientists and food regulators must also stay aware of developments in the food supply and importantly consider the impact on consumers. The end focus for all stakeholders is food purchase, but with varying interests, be it health profiles, product sales or food consumption. Whether and how health claims influence consumer decisions is highly relevant to regulators as they work to protect public health and safety. To do this, however, there must also be uptake from food manufacturers, and an agreed process for managing claims. This paper examines the issue of the translation of science within N&HC by reviewing the impact of health claims on consumer behaviour and identifying issues that may arise from claims regulation in Australia.

## How Do Consumers Interact with Nutrition and Health Claims on Foods?

Increasing numbers of studies have explored the influence of nutrition and health claims on consumer perceptions of products and the likelihood of purchasing or consuming products. For example, in one meta-analysis [10], products carrying a claim were significantly more likely to be purchased or consumed than an identical product without a claim (odds ratio: 1.75, 95% confidence intervals: 1.60–1.91). Interestingly, both nutrition content (odds ratio: 1.74, 95% confidence intervals: 1.29–2.35) and health claims (odds ratio: 1.73, 95% confidence intervals: 1.57–1.91) seemed to have similar effects on the likelihood of product purchasing or consumption. This suggests that, overall, N&HC may influence the way consumers discriminate between products, but the nutrition concepts underpinning different types of claims may not be appreciated at point of purchase. On the other hand, holistic health reasons rather than simple nutrient delivery may influence intention to purchase. In an analysis of some 24 studies [11], consumers expressed positive views towards products that contained all types of claims, but lent more toward high level health claims than general level health claims or nutrition content claims. Further research may be informative of how these distinctions play out, but either way, it appears that N&HC represents an opportunity to influence public health and the quality of food supply.

More specifically, claims relating to energy and fat content may be of particular public health interest, given the context of global obesity [12]. This is an example where total diet is paramount, but individual foods can differ widely in their contributions to excessive intakes. In addition, while the concept of total energy intake could be seen as relatively straightforward, the science behind dietary fat is quite complicated and presents with real challenges for translation [13]. One review [14] found varying degrees of influence on food choice from energy and fat claims. While there was some evidence that these claims resulted in decreased consumption of energy-dense, nutrient-poor foods, or increased consumption of nutrient-dense foods, other studies in the review found either no or undesirable impacts on consumption. Similarly, Oostenbach et al. [15••] examined the evidence on the effect of nutrition content claims (specifically those related to fat, sugar and energy) on food choices and energy intake. Overall, products containing nutrient content claims were considered to be healthier and have a lower energy content than products without claims. Nutrient content claims could influence purchase intentions and increase consumption, although these effects did vary based on product and claim type.

At this stage, it appears that N&HC can influence consumer purchasing and consumption of food but more research is required on how specific claims may be interpreted and actioned by consumers. Addressing the concepts behind the claims and their relevance to dietary patterns may be a good start.

## How Do Regulators and Manufacturers Work with N&HC?

The regulatory system is a major interface for addressing the impact of food consumption on health. From a public health perspective, it provides a vehicle for communicating the benefits of foods to assist in food choices, but this action is multifaceted and involves many key stakeholders in the food system. The ongoing evolution of N&HC in Australia attests to the processes and challenges which need to be addressed. In the first instance, while the lack of mandatory regulations on N&HC was recognised as a potential risk to public health and safety [16], a substantial amount of work was required over many years to develop a standard. Following a discussion and concept paper developed by the Australia New Zealand Food Authority (ANZFA, the predecessor of FSANZ) in 1993 and 1996, respectively [17], and a 2004 proposal (Proposal P293) [18], the Food Standards Code was amended. The updated Standard 1.2.7 (Nutrition, Health, and Related Claims) was gazetted in 2013, becoming mandatory in 2016 [6], some 20 years after the original concept paper.

Ongoing research continues to inform the development of the system. For example, a number of studies have explored the use of N&HC by manufacturers for selected food categories. In the Illawarra region, south of Sydney, Sussman et al. [19] conducted a cross-sectional audit of N&HCs on breakfast cereal products in supermarkets and found that of the 329 products audited, 315 (95.7%) carried at least one claim, and the majority of claims were nutrition content claims. Later, Wadhwa et al. [20] conducted a similar audit of dairy yoghurts, with 97.9% of products carrying at least one claim, predominantly nutrition content claims. Despite some differences in methodology, this was an increase in the use of claims found in a national study conducted by Pulker et al. prior to Standard 1.2.7 [21]. In this earlier study, Pulker et al. gathered data from both websites and physical stores and found only 59% of products (ultra-processed foods including breakfast cereals, confectionary, and snacks) carried nutrition or health claims. A Sydney-based supermarkets study by Wellard-Cole et al. [22], auditing claims on non-alcoholic beverages, cereal bars and breakfast cereals in 2011 and again in 2016, confirmed that change was happening (increasing from 67% of audited products in 2011, to 76% in 2016, *p* < 0.001). Of the 1737 products audited in 2016, 76% carried at least one claim, the majority (82%) being content claims [22]. Thus, there appeared to be a high prevalence of claims on foods in Australian supermarkets, with increasing use over time, suggesting a high acceptance of the system by manufacturers. Nevertheless, concerns have continued to be expressed on the potential to mislead consumers [22], who may attribute broader health benefits to products on the basis of their content claims [23], especially with the high use of content claims reported in the literature [19–22].

Potential limitations have also been highlighted in the avenues by which claims are approved. Within Standard 1.2.7, there are a number of pre-approved general and high level health claims which can be made, dependent on a food meeting the NPSC and specific conditions such as minimum nutrient levels [6]. In the case of general level health claims however, food businesses may also self-substantiate a food-health relationship via a process involving a systematic review [6]. Following completion of the systematic review, the business notifies FSANZ of their substantiated food-health relationship [6, 24], and if they meet the requirements of Standard 1.2.7, may then make the claim. The regulatory frameworks of a range of countries include processes allowing for consideration of food-health relationships which are not currently pre-approved [3–5]. It should be noted, however, that under the current Australian and New Zealand system, FSANZ performs the function of the standard setting body [24]. Compliance with the Food Standards Code is the responsibility of the State and Territory authorities and the New Zealand government, so in a case where a complaint is made, these authorities evaluate the systematic review supporting a self-substantiated general level health claim. This process also places the onus for accurately substantiating the review on the food business, a potential limitation of the current process [25••, 26]. However, in New Zealand, claims which have been notified to FSANZ are formally reviewed by the New Zealand Ministry for Primary Industries. This involves a process of evaluating the systematic review underpinning the food-health relationship [27]. If the New Zealand Ministry for Primary Industries finds that the food-health relationship cannot be substantiated, food businesses must request FSANZ remove their notified relationship. This process ensures a level of regulatory oversight, which has been criticized as lacking in the Australian process [25••].

Recently, Wellard-Cole et al. [25••] explored the robustness of the self-substantiation process by monitoring food-health relationships notified to FSANZ between 2013 and 2017. Of the 63 food-health relationships notified by Australian companies during this time, only 52% were considered by the study authors to have adequate published evidence to substantiate the relationship. A total of 27 food-health relationships were determined to have equivocal or insufficient evidence and were subsequently referred to authorities, resulting in several relationships being removed from the FSANZ website. These findings expose significant problems with the current framework for self-substantiation of general level health claims. While the situation may vary across global jurisdictions, pre-approval of food-health relationships may be required before claims can be made recommended.

## Future Directions

It appears both nutrition content and health claims may generally influence consumer’s purchasing, consumption and overall perceptions of a product, but it is unclear how these work more specifically in terms of targeted changes to dietary patterns. Likewise, the Australian experience suggests N&HC are being taken up by manufacturers, but there are issues with the management of evidence review. From a nutrition perspective, both these problems are related to the translation of science, how it is applied, communicated, understood and acted upon [26]. The ongoing development of regulatory frameworks need to address these issues to assure a robust system that supports consumers to make informed and healthy choices.

After almost 30 years, the Australian FSANZ Act is currently under major review [28]. The options under consideration include maintaining the status quo; modernising the Act to make it agile, resilient and fit for purpose; and extending the role of FSANZ. Modernising includes a component of risk-based approaches to developing and amending food regulatory measures. There is recognition of evolving new technologies, global supply chains, food innovation and shifts in dietary patterns and consumer expectations. Importantly the value of leveraging food regulation to influence dietary patterns toward better health remains. Addressing the issues raised in this review will need to occur in this revised context.

New ways of thinking around consumer involvement have also emerged in the literature. Rather than simply test consumer opinions, there is strong argument for engaging consumers more directly through their ‘lived experiences’ [29]. There may be a need to rethink the way N&HC are constructed, not just in wording (which strongly privileges a scientific discourse), but also in terms of the contexts in which foods are purchased and consumed. In addition genuine efforts to improve the nutrition science literacy of the population may assist in establishing greater connections with the type of information that is currently available on labels. The findings that consumers appear to value N&HC is promising. Even so, current N&HC and associated tools, such as the Nutrient Profiling Scoring Criterion, need to keep up to date with emerging evidence of the diet-disease relationship. Ways need to be found of evaluating individual foods in terms of contributions to dietary patterns, to better reflect the interdependence of foods and total diets and communicate that to consumers.

The science of evidence evaluation in nutrition which underpins N&HC is also constantly evolving [26, 30•]. Associated new methodologies and technologies need to be taken up in a fit for purpose food regulation system. Up to date food composition data and dietary intake surveys are fundamental components of the risk assessment associated with food regulation. Even food categorisation is under review, with a closer eye now aimed toward degree of processing [31, 32]. Models for assessment of N&HC could be developed that focus on health priorities and are more pro-active rather than reactive in relation to food innovation and associated claims. Efficiencies in evidence review could be achieved by integrating efforts with other areas associated with food policy, such as national dietary guidelines. Finally building nutrition science and communications capacity in food regulation seems essential to manage and assist all levels of operations, including businesses keen to use the N&HC opportunities. This may also help to ensure compliance and reduce the burden on enforcement agencies which are required to investigate complaints.

There are risks associated with change that should also be considered. For example, presently in Australia, high level health claims may not be self-substantiated; only pre-approved claims are allowed. However, food companies may apply to change the Food Standards Code, through a formal process evaluated by FSANZ, which includes a systematic review of the proposed food-health relationship. As part of the FSANZ Act review, it is has been proposed that this pathway be abolished due to limited uptake, but opportunities may be lost, for example, if dietary guidelines go another way [28]. These issues may also be relevant to other jurisdictions across the globe.

## Conclusions

Nutrition and health claims can play a significant role in driving consumer choices towards a healthier food supply. As part of the food regulation system, however, they fit within a complex interplay between multiple groups of stakeholders. Nutrition science underpins the public health agenda and informs the development of N&HCs, but a broader sensitivity to consumer understandings of nutrition and their lived experiences with food may be required. As an example, the Australian experience with N&HC continues to evolve, reflecting developments in methodologies and a fundamental appreciation of the relationship between food and health, positions which are universal to all jurisdictions.
